# Twelve ESMO Congress 2022 breakthroughs: practicing oncologists’ perceptions and potential application on presented data

**DOI:** 10.1016/j.esmoop.2022.100773

**Published:** 2023-01-10

**Authors:** H.K. van Halteren, J. Bennouna, B. Brasiuniene, A. J. Cunquero Tomas, A. M. Garcia Trinidad, A. Indini, G. Liposits, B. Pellegrino, L. Popovic, A. Tan, R. Vidra, M. Strijbos, Practising Oncologists Working Group

**Affiliations:** 1 Department of Medical Oncology, Adrz Hospital, Goes, The Netherlands; 2 Department of Medical Oncology, Hospital Foch, Suresnes, France; 3 Department of Medical Oncology, National Cancer Institute of Lithuania, Faculty of Medicine, Vilnius University, Vilnius, Lithuania; 4 Department of Medical Oncology, General University Hospital of Valencia, Valencia, Spain; 5 Medical Oncology Unit, General Hospital of Requena, Valencia, Spain; 6 Section of Medical Oncology, Dagupan Doctors Villaflor Memorial Hospital, Dagupan City, The Philippines; 7 Unit of Medical Oncology, Department of Oncology, Ospedale di Circolo e Fondazione Macchi, ASST Settelaghi, Varese, Italy; 8 Department of Clinical Research, University of Southern Denmark, Odense, Denmark; 9 Medical Oncology and Breast Unit, University Hospital of Parma, Parma; 10 Department of Medical Oncology, University of Parma, Parma, Italy; 11 Oncology Institute of Vojvodina, Faculty of Medicine, University of Novi Sad, Novi Sad, Serbia; 12 Department of Medical Oncology, Waikato Hospital, Hamilton, New Zealand; 13 Department of Medical Oncology, Regional Institute of Gastroenterology and Hepatology “Prof. Dr. Octavian Fodor”, Cluj-Napoca, Romania; 14 UBBMed, Babes-Bolyai University, Cluj-Napoca, Romania; 15 Department of Medical Oncology, GZA Hospitals, Wilrijk, Belgium

**Keywords:** ESMO Congress 2022, review, methodology, implementation

## Abstract

**Background:**

During the European Society for Medical Oncology (ESMO) Congress 2022, outcome data of a great number of clinical trials were presented. For the attending medical oncologist, it is important to structure these data in a way that facilitates a trade-off between treatment burden and benefit.

**Materials and methods:**

To illustrate this, we carried out a narrative non-systematic review of 12 selected oral presentations with potential impact on future daily practice, focusing on trial methodology, possible study flaws, reported clinical benefit and implementability.

**Results:**

The selected presentations encompassed 10 phase III trials, 1 randomized phase II trial and 1 phase II trial. In 7 out of 12 trials, quality of life and/or patient-reported outcomes had been evaluated. None of the trials, which reported progression-free survival (PFS) data, provided information, which could exclude informative censoring bias. In none of the trials reporting overall survival (OS) data, potential flaws due to undesirable crossover and imbalance between study groups regarding post-progression treatments were addressed. For the 11 reviewed randomized trials, the ESMO-Magnitude of Clinical Benefit Scale (MCBS) grade achieved with the new intervention was calculated based on the presented data. The MCBS grade varied from 1 to 5.

**Conclusions:**

Our review confirms the high-quality standard of current cancer research and the clinical relevance of the research questions answered. However, during presentation of PFS and/or OS data, factors known to affect PFS and OS analysis should be structurally addressed. In order to keep cancer care affordable and sustainable, it could be considered to include an ESMO-MCBS threshold in the drug appraisal process of regulatory authorities.

## Highlights

• During the ESMO Congress 2022 outcome data of a great number of trials were presented.
• Data appraisal requires a trade-off between treatment burden and benefit.
• To illustrate this we carried out a narrative non-systematic review of 12 selected oral presentations.
• Factors known to affect PFS and OS analysis were not structurally addressed.
• Selection of only fit patients in later-line advanced cancer trials may limit generalizability.

## Introduction

During the European Society for Medical Oncology (ESMO) Congress 2022, outcome data of a great number of trials were presented, some of which are expected to change daily clinical practice (for list of abbreviations, see Table 1). For the attending medical oncologist, it is important to structure this vast amount of data in a way that facilitates a trade-off between treatment burden and benefit. Key questions are:

1. Are the conclusions made supported by the presented data?
2. How robust are the data presented, from the perspectives of trial methodology and potential flaws?
3. Which benefit could the average cancer patient derive from the new treatment in real-world settings?
4. Is this benefit sufficient to justify adoption of the new treatment in daily practice as standard of care?

**Table 1:** List of abbreviations used in the manuscript

DFS	Disease-free survival
ESMO	European Society for Medical Oncology
GHS	Global health status
ICI	Immune checkpoint inhibitor
OS	Overall survival
ORR	Overall response rate
MCBS	Magnitude of Clinical Benefit Scale
NSCLC	Non-small-cell lung cancer
PF	Physical functioning
PRO	Patient-reported outcome
PS	ECOG performance status
QoL	Quality of life
TPC	Treatment according to physicians’ choice
TRAE	Treatment-related adverse event
TTD	Time to deterioration

In order to clarify this thinking process, we carried out a narrative non-systematic review of 12 selected oral presentations with potential impact on future daily practice, focusing on trial methodology, possible study flaws, reported clinical benefit and implementability. The review is structured according to previously proposed potential bias items, which could distort the appraisal of clinical benefit.^1^ Furthermore, the clinical benefit reported is graded in terms of the ESMO-Magnitude of Clinical Benefit Scale (MCBS).^2^

## Materials and methods

The 12 members of the ESMO Practising Oncologists Working Group were subdivided into 6 groups, based on their specific expertise. Each expertise group was assigned with the task to select two oral presentations of phase II and/or phase III data for one of the following six disease items: (i) breast oncology, (ii) thoracic oncology, (iii) urologic oncology, (iv) gastrointestinal oncology, (v) gynecologic oncology and (vi) innovative treatments. The ESMO Congress 2022 abstracts and presentations, as well as the trial information provided at www.clinicaltrials.gov, were used for the reviewing process, which focused on the following items:

I. With regard to the basic trial structure:

Could the patients included in the trial be regarded as daily practice patients?

Could the treatment given in the comparator arm be regarded as an accepted gold standard?

Was randomization blinded?

Was the trial discontinued prematurely based on Early Stopping Rules?

II. With regard to data presentation:

Was the risk of censoring bias clarified in relation to presented surrogate endpoints, such as recurrence-free and progression-free survival (PFS)?

Were overall survival (OS) data underlined with information regarding undesirable crossover and imbalances between study groups with regard to post-progression treatments?

III. With regard to reported clinical benefit:

Which benefit is achieved with the new intervention compared to the study control arm? For all randomized trials reviewed, we calculated the ESMO-MCBS grade. For the scoring process, the ESMO-MCBS V1.1—Evaluation Forms were used.^2^

## Breast oncology

### TROPICS-02

TROPICS-02 is an open-label, randomized, prospective, multicenter, phase III study evaluating the efficacy of sacituzumab govitecan (SG) compared to treatment of physician’s choice (TPC) in patients with locally advanced/metastatic, hormone receptor-positive, human epidermal growth factor receptor 2 (HER2)-negative breast cancer after failure of at least two—but no more than four—prior chemotherapy regimens for metastatic disease.^3^ Previous treatment with at least one endocrine therapy, a taxane and a cyclin-dependent kinase 4/6 inhibitor in any setting was a required criterion for inclusion in this trial. Crossover was not allowed. The primary endpoint was PFS, and key secondary endpoints included objective response rate (ORR), OS and quality of life (QoL). The European Organisation for Research and Treatment of Cancer (EORTC) Core Quality of Life questionnaire (QLQ-C30) was used to measure the patients’ global health status (GHS), fatigue and pain over time (scoring range 0-100). For these three items, the threshold for a minimal clinically important difference (MCID) was defined as at least 10 points worsening from baseline.^4^ In total, 543 patients were randomized in a 1 : 1 ratio to receive SG (*n* = 272) versus TPC (*n* = 271). The number of patients who did not receive the allocated therapy was considerably higher in the TPC arm (22 versus 4 patients), but there were no other imbalances across the two treatment groups. The ORR was higher in the SG study arm (21% versus 14%), as well as the PFS [hazard ratio (HR) of progression 0.66, 95% confidence interval (CI) 0.53-0.83]. However, these results should be interpreted with caution due to the imbalance in informative censoring between the two groups (e.g. the percentage of patients censored at 6 months was 24% in the SG arm and 35% in the TPC arm). The SG arm was also superior in terms of OS (HR 0.79, 95% CI 0.65-0.96). Data on post-progression treatment were however not presented and an imbalance between treatment arms can therefore not be excluded. More patients experienced serious treatment-related adverse events (TRAEs) (i.e. grade 3 or more according to the Common Terminology Criteria for Adverse Events, CTACE) in the SG arm (74% versus 60%). Among six TRAEs leading to death, only one was considered as treatment-related (septic shock due to neutropenic colitis). In the SG arm, the median time to deterioration (TTD) was superior with regard to GHS (4.3 versus 3.0 months) and fatigue (2.2 versus 1.4 months).^5^ The TTD with regard to pain was similar in both treatment groups. Based on the data presented (HR of death 0.79, improved QoL, median OS in the comparator group <12 months), the MCBS grade achieved with SG is 2.

## MonarcHER

This open-label, three-arm, randomized, phase II trial was carried out in patients with advanced hormone receptor-positive, HER2-positive breast cancer, who had received at least two prior HER2-directed therapies—including trastuzumab emtansine (T-DM1)—and a taxane. Only patients with a performance status (PS) of 0 or 1 were eligible. Overall, 237 patients were randomly assigned (1 : 1 : 1) to receive abemaciclib/trastuzumab/fulvestrant (study arm A), abemaciclib/trastuzumab (study arm B) or trastuzumab/investigators’ choice chemotherapy (study arm C).^6^ Primary endpoint was PFS, and secondary endpoints included ORR, OS and QoL. PFS data had been published previously, showing better PFS results for arm A compared with arm C (HR 0.67, 95% CI 0.45-1.00), while study arm B showed non-inferiority.^7^ In the ESMO 2022 update, the authors presented the OS data. Neither study arm A nor study arm B showed a statistically significant OS benefit compared to study arm C. This observation could be due to the fact that the study was not sufficiently powered to ascertain a difference in OS. Based on the data presented (HR of progression 0.67, median PFS in the comparator group <6 months), the MCBS grade achieved with the combination of abemaciclib, fulvestrant and trastuzumab is 1.

## Thoracic oncology

### CodeBreak 200

The CodeBreak 200 trial is an open-label, randomized, phase III study comparing the efficacy of K*ras* inhibitor sotorasib (experimental arm) with docetaxel (standard arm) in patients with K*ras*G12C-mutated advanced non-small-cell lung cancer (NSCLC) pre-treated with platinum-based chemotherapy combined with an immune checkpoint inhibitor (ICI).^8^ Overall, 345 patients were randomized in a 1 : 1 ratio to treatment with sotorasib (*n* = 171) or to treatment with docetaxel (*n* = 174). The primary endpoint was PFS and secondary endpoints included OS, ORR, safety and QoL. The EORTC QLQ-C30 questionnaire was used to measure the TTD for GHS (CMID threshold ≥8 points worsening from baseline), physical functioning (CMID threshold ≥13 points worsening from baseline), cough, chest pain and dyspnea (crude CMID thresholds of 67, 67 and 92 points, respectively).^9^ During patient enrolment, a protocol amendment reduced the number of patients needed from 650 to 330 patients, and authorized crossover from docetaxel to sotorasib. This amendment was not further explained. ORR was significantly higher with sotorasib (28.1% versus 13.2%, *P* < 0.001), and PFS was superior as well (HR 0.66, 95% CI 0.51-0.86). An imbalance in informative censoring cannot be excluded, since censoring data were not presented. OS was not statistically different between the two treatment arms, with a median OS of 10.6 months with sotorasib versus 11.3 months with docetaxel (HR 1.01, 95% CI 0.77-1.33). Thirteen patients (7.5%) in the standard treatment arm received sotorasib after progression. This undesirable crossover could have affected the OS analysis. The PFS and OS data reported in the docetaxel arm were better than could be expected from previous studies. Serious TRAEs occurred more often in the sotorasib study arm than in the docetaxel study arm (40.4% versus 33.1%), with one and two treatment-related deaths with sotorasib and docetaxel, respectively. The median TTD with regard to GHS and physical functioning was superior in the sotorasib arm. Based on the data presented (HR of progression 0.66, median PFS in the comparator arm <6 months, improved QoL), the MCBS grade achieved with sotorasib is 2.

### IPSOS

In this open-label, randomized, phase III trial, patients with advanced/recurrent treatment-naive NSCLC without EGFR or ALK sensitizing mutations, who were not considered fit enough to receive platinum-doublet chemotherapy (i.e. elderly, PS 2-3, comorbidities), were allocated in a 2 : 1 ratio to receive atezolizumab monotherapy [1200 mg intravenously (i.v.) q3w] or investigators’ choice chemotherapy (e.g. vinorelbine, gemcitabine).^10^ Crossover was not allowed. The primary endpoint was OS, and secondary endpoints included ORR, PFS, safety and QoL, as assessed by the QLQ-C30 and the Quality of Life Questionnaire-Lung Cancer 13 (QLQ-LC13). For all items scored, the CMID threshold was defined as at least 10 points worsening from baseline. OS was superior in the atezolizumab study arm (HR 0.78, 95% CI 0.63-0.97), with a median OS benefit of 1.1 month. The OS analysis could have been affected by significant imbalances between the intervention and the comparator group with regard to post-progression treatments. The percentage of patients who subsequently received chemotherapy was higher in the intervention group (15.9% versus 10.6%), whereas considerably more patients in the comparator group received subsequent immunotherapy (18.5% versus 1.3%). ORR was superior in the atezolizumab study arm (16.9% versus 7.9%), whereas stable disease was more often accomplished in the chemotherapy study arm (48.3% versus 40.4%) and there was no difference in PFS. The toxicity profile favored atezolizumab, with less serious TRAEs (16.3% versus 33.3%), less toxic deaths (1% versus 2.7%) and a treatment discontinuation rate comparable across treatment groups (13% versus 13%). The TTD with regard to chest pain was significantly longer in the atezolizumab study arm (HR of deterioration 0.51, 95% CI 0.27-0.97), but for all the other patient-reported outcome (PRO) measures no difference between the two study arms was found. Based on the data presented (HR of death 0.78, median OS in the comparator group <12 months, significantly less serious TRAEs), the MCBS grade achieved with atezolizumab is 2 in the first-line setting of patients who are not considered fit enough to undergo more intensive treatment due to older age, PS ≥2 and/or comorbidities. However, it should be underlined that PS has a stronger prognostic impact than older age, as was shown in a recently published real-world study on first-line monotherapy with ICIs.^11^ Median PFS in patients with PS ≥2 was 2.7 months compared to 4.3 months in patients aged 75 years or older. Considering such poor figures, best supportive care remains an alternative to be discussed.

## Urologic oncology

### COSMIC-313

COSMIC-313 is a double-blinded, randomized, phase III study in previously untreated patients with advanced intermediate- or poor-risk clear-cell renal cell cancer (RCC).^12^ All patients were randomized (1 : 1) to receive immunotherapy with 3-weekly nivolumab (Nivo, 3 mg/kg i.v.) and 3-weekly ipilimumab (Ipi, 1 mg/kg i.v.) for four cycles, followed by 4-weekly cycles of Nivo (480 mg i.v.), for up to 24 months, plus cabozantinib 40 mg once daily (C) or placebo (P). Randomization was stratified by International Metastatic RCC Database Consortium (IMDC) risk groups and by region. The standard arm of Nivo/Ipi could be considered as a current standard-of-care option for patients with newly diagnosed IMDC intermediate-/poor-risk clear-cell RCC. The primary endpoint was PFS by blinded independent radiology in the first 550 randomized patients (progression-free survival Intention To Treat population). The secondary endpoint was OS in all randomized patients (intention-to-treat population); additional endpoints included ORR and safety outcomes. A total of 885 patients were randomized (428 patients in the C arm and 427 patients in the P arm). ORR was superior in the experimental arm (40.3% versus 36%). PFS also appeared superior in the experimental arm (HR 0.73, 95% CI 0.57-0.94). However, looking into the PFS graphs, there is a difference in events between the two study groups of only 17, and a far larger difference in patients at risk over time, suggesting that more patients were point censored in the control group, which could have led to censoring bias. In the subgroup analysis, the reported PFS benefit appeared to be limited to the intermediate-risk patients’ group (HR 0.63, 95% CI 0.47-0.85), which made up 75% of the study population. In the poor-risk group, the HR for progression was 1.04 (95% CI 0.65-1.69). No OS data were reported. Serious TRAEs occurred more often in the C arm (73% versus 41%). Discontinuation of all treatment drugs due to TRAEs occurred more often in the C arm as well (12% versus 5%). Based on the data presented (HR of progression 0.73, median PFS in the comparator group >6 months), the MCBS grade achieved with the addition of cabozantinib is 1.

### CheckMate 914

The CheckMate 914 trial was a prospective, randomized, double-blinded, placebo-controlled, phase III trial, which estimated the efficacy of adjuvant therapy with Ipi and Nivo after nephrectomy for localized RCC with a high risk of relapse (i.e. pT2a/G3-4, pT2b-4/any grade, N1/any T, any grade).^13^ Patients were randomly assigned in a 1 : 1 ratio to receive 24 weeks of immunotherapy or placebo. Primary endpoint was disease-free survival (DFS). Secondary endpoints were OS and TRAEs. Overall, 405 patients received Ipi/Nivo and 411 patients received placebo. There was no difference in DFS between the study arms. Serious TRAEs occurred more often in the immunotherapy arm (28% versus 2%). Forty-three percent of patients discontinued immunotherapy, the majority because of side-effects. With regard to daily practice, the question is whether it is prime time for adjuvant immunotherapy in RCC patients with a high risk of relapse. Whereas adjuvant treatment with Ipi/Nivo (CheckMate 914) or atezolizumab (IMmotion010 trial, results also presented at ESMO 2022) did not result in an improved DFS, the KEYNOTE-564 trial has shown a DFS benefit after adjuvant treatment with pembrolizumab (HR of progression 0.63, 95% CI 0.50-0.80).^14^^,^^15^ Perhaps, immunotherapy performs better in the neoadjuvant or perioperative setting and there are many studies ongoing to address this issue. Single-agent studies include investigation of neoadjuvant pembrolizumab (NCT02212730), neoadjuvant Nivo (NCT02575222) and three perioperative trials (NCT02595918, NCT02595918 and NCT03055013). Combination strategies under investigation are neoadjuvant durvalumab plus tremelimumab (NCT02762006), neoadjuvant spartalizumab plus canakinumab (NCT04028245), neoadjuvant axitinib plus toripalimab (NCT04118855), neoadjuvant axitinib plus avelumab (NCT03341845) and neoadjuvant sitravatinib plus Nivo (NCT03680521).

## Gastrointestinal oncology

### HR-IRI-APC

HR-IRI-APC is a prospective, randomized, controlled, double-blinded, phase III trial, which compared the efficacy of 5-fluorouracil/leucovorin (5-FU/LV) combined with HR070803 (a liposomal formulation of irinotecan) versus 5-FU/LV combined with placebo in the second-line treatment setting of patients with locally advanced or metastatic pancreatic cancer, who had progressed on gemcitabine-based therapy. The primary endpoint was OS. Secondary endpoints were ORR, PFS and safety.^16^

Two hundred and ninety-eight patients were randomly assigned in a 1 : 1 ratio to receive either HR070803 56.5 mg/m^2^ plus 5-FU/LV 2000/200 mg/m^2^ (arm A) or placebo plus 5-FU/LV 2000/200 mg/m^2^ (arm B). At a median follow-up of 12.8 months, the primary endpoint was met with a median OS of 7.4 months (95% CI 6.0-8.4 months) in arm A and a median OS of 5.0 months (95% CI 4.3-6.0 months) in arm B (HR of death 0.63, 95% CI 0.48-0.84). The OS data presentation however did not encompass information regarding desirable crossover, undesirable crossover and post-progression treatments per study group. ORR was higher in study arm A (13% versus 0.7%), which also showed a PFS benefit (HR of progression 0.36, 95% CI 0.27-0.48). Based on the PFS data presented, informative censoring cannot be ruled out. Serious TRAEs occurred more often in study arm A (53.1% versus 43.1%). Based on the data presented (HR of death 0.63, median OS in the comparator group <12 months, median OS gain between 2 and 3 months), the MCBS grade achieved with the addition of HR070803 is 3. However, in order to translate these trial data into a clinical practice guideline, it would be important to know why these very fit (PS 0-1) patients had not received FOLFIRINOX treatment in first line.

### NICHE-2

The NICHE-2 trial is a multicenter, phase II study which explored the efficacy of Nivo in the neoadjuvant setting of mismatch repair-deficient (dMMR) colon cancer.^17^

Before resection, patients with cT2-4 and/or N1-2 dMMR colon cancer received a single dose of Ipi (1 mg/kg) and two doses of Nivo (3 mg/kg). Resection was carried out within 6 weeks after the first treatment cycle. Co-primary endpoints were safety and 3-year DFS; secondary endpoints included major pathological response (MPR) defined as residual viable tumor ≤10% and complete response [pathological complete response (pCR)] rates. The trial comprised 112 patients (58% female) with a median age of 60 years (range 20-82 years). All patients had a PS 0-1, 77% had high-risk disease (cT4 and/or cN2) and 68% of the primary tumors were right-sided. Thirty-one patients had Lynch syndrome. The median time from the first ICI dose to surgery was 5.4 weeks, only 2% of patients had delayed surgery due to adverse events (AEs) and all patients underwent an R0 resection. Any pathological response was observed in 99% of patients, of whom 95% had MPR including 67% pCR. Grade 3-4 AEs were observed in 3% of patients. None of the patients experienced disease recurrence after a median follow-up of 13 (range 1-57) months. With regard to daily practice, neoadjuvant treatment with Ipi and Nivo could radically change the treatment paradigm in (borderline) resectable dMMR colon cancer. Unfortunately, the prevalence of dMMR is lower in left-sided colon and rectal cancer. From a general standpoint, the NICHE study data pose the question whether ICI therapy would be more effective in the neoadjuvant setting than in the adjuvant setting, due to a higher abundance of antigenic stimuli.

## Gynecologic oncology

### 309/KEYNOTE-775

The 309/KEYNOTE-775 trial is an open-label, randomized, phase III study which compared the efficacy of lenvatinib and pembrolizumab (LenPembro) versus TPC in the second-line setting of advanced, metastatic or recurrent endometrial cancer.^18^ Patients had received one prior platinum-based chemotherapy in first line (up to two if one was given in the neoadjuvant/adjuvant setting) and were randomized in a 1 : 1 ratio to receive either LenPembro or TPC (doxorubicin or paclitaxel). Crossover was allowed. Patients were stratified by mismatch repair (MMR) status; patients with proficient (p)MMR tumors were further stratified by PS, geographic region and history of pelvic irradiation. Primary endpoints were PFS and OS. Secondary outcomes were ORR, QoL, TRAEs and pharmacokinetics. Following previous publication, updated outcome measures were presented.^19^ Both ORR (33.8% versus 14.7%) and PFS (HR of progression 0.56, 95% CI 0.48-0.66) were superior in the LenPembro arm. Based on the PFS data presented, informative censoring cannot be ruled out. OS was also superior in the LenPembro arm (HR of death 0.65, 95% CI 0.55-0.77) and the same benefit was shown in the (p)MMR subgroup. Data on post-progression treatment per treatment group were not presented, but undesirable crossover was reported with 8.7% of patients in the TPC group receiving LenPembro in third line. After excluding these patients, the HR of death was 0.60 (95% CI 0.51-0.71). The percentage of serious TRAEs was lower in the TPC arm (60.1% versus 78.8%). Based on the data presented (HR of death 0.65, median OS in the comparator group between 12 and 24 months, median PFS gain >5 months), the MCBS grade achieved with LenPembro is 4. Regarding daily practice, LenPembro appears a valuable new treatment option in second line, regardless of the MMR status. But the majority of included patients were in a remarkably good clinical condition [60% Eastern Cooperative Oncology Group (ECOG) PS 0, 40% ECOG PS 1], which is often not the case in daily practice, and nevertheless lenvatinib had to be discontinued in 35.7% and pembrolizumab in 22.2% of patients. It would be wise to offer the LenPembro option only to patients with an ECOG PS <2.

### SOLO1/GOG-3004

The phase III SOLO1/GOG-3004 trial was conducted to evaluate the efficacy of maintenance therapy with olaparib in patients with BRCA-mutated newly diagnosed International Federation of Gynecology and Obstetrics (FIGO) stage III/IV high-grade serous or endometrioid ovarian, primary peritoneal and/or fallopian tube cancer, who had achieved a complete or partial response to platinum-based chemotherapy without bevacizumab.^20^ Patients were randomized in a quadruple blinding fashion (participant, care provider, investigator, outcome assessor) and in a 2 : 1 ratio to receive either olaparib 300 mg twice daily for up to 3 years or until disease progression (*n* = 260) or placebo (*n* = 131). Primary endpoint was PFS; secondary endpoints were OS and safety. QoL was not assessed. The most recent analysis on PFS, which was carried out in March 2020, revealed a significantly improved HR of progression (0.33, 95% CI 0.25-0.43).^21^ Now the OS data were presented, which showed a clear improvement as well (HR of death 0.55, 95% CI 0.40-0.76). Post-progression treatments were specified per study group, but 44.3% of patients in the placebo group were reported to have received subsequent poly (ADP-ribose) polymerase (PARP) inhibitor therapy. Serious TRAEs occurred more often in the olaparib study group (39.6% versus 20%), but there were no treatment-related deaths in both study arms. Based on the data presented (HR of death 0.55, median OS in the comparator group >24 months, median OS gain >9 months), the MCBS grade achieved with olaparib maintenance is 4 points. With regard to daily practice, these data highlight the importance of routinely carrying out BRCA mutation testing and offering first-line PARP inhibitor maintenance therapy to all patients with advanced BRCA mutation-positive disease rather than delaying until recurrence in order to achieve long-term remission. Considering the entire treatment sequence, the question is whether the addition of bevacizumab to primary treatment and maintenance therapy offers additional benefit. In this respect, the presented OS data of the PAOLA-1/ENGOT-ov25 trial should be mentioned.^22^ This trial comprised the same case mix and also questioned the efficacy of olaparib maintenance therapy, but in the comparator arm patients had received primary and maintenance therapy with bevacizumab. Whereas 5-year OS percentages in the olaparib arms of SOLO-1 and PAOLA-1 were comparable (73.1% versus 73.2%), the bevacizumab-free comparator arm appeared to outperform the comparator arm including primary and maintenance therapy with bevacizumab (63.4% versus 53.8%). Furthermore, the percentage of serious TRAEs was clearly lower as well (20% versus 51%).

## Innovative treatments

### DeFi

The DeFi trial is a phase III, randomized, double-blinded, placebo-controlled trial of nirogacestat, a γ-secretase inhibitor, for progressing desmoid tumors (DTs).^23^ The trial included adult patients with treatment-naïve DT not amenable to surgery, or with refractory/recurrent disease after at least one line of systemic therapy. Overall, 142 patients were randomized in a 1 : 1 ratio to receive nirogacestat 150 mg twice daily versus placebo, with the option of open-label nirogacestat access for patients in the placebo arm demonstrating progressive disease. The primary endpoint was PFS; secondary endpoints included ORR and PRO (Brief Pain Inventory—Short Form and GODDESS-DT symptom score). ORR (41% versus 8%) and PFS (HR of progression 0.29, 95% CI 0.15-0.55) were superior in the nirogacestat arm. Based on the PFS data presented, informative censoring cannot be ruled out. The percentage of serious TRAEs in the nirogacestat arm was higher (57% versus 17%), but nirogacestat reduced pain and overall symptom severity, while sustaining physical and role functioning. Based on the data presented (HR of progression 0.29, median PFS in the comparator group >6 months, median PFS gain >3 months, improved QoL, long-term plateau in the PFS curve), the MCBS grade achieved with nirogacestat is 5. Further research is needed to demonstrate whether this new drug will perform better than first-line standard systemic treatment as upfront therapeutic strategy.

### TILs in melanoma

In two specialized cancer centers, a randomized, prospective, phase III trial was carried out in patients with stage IIIC/IV melanoma (NCT02278887) to compare the efficacy of a single administration of interleukin 2-primed tumor-infiltrating lymphocytes (TILs) after lymphocyte-depleting therapy (intervention arm) with four 3-weekly cycles of Ipi (comparator arm).^24^ Treatment-naïve patients and patients who had received one previous adjuvant or palliative systemic treatment (excluding Ipi) were eligible. Primary endpoint was PFS. Secondary endpoints were immune-related PFS and safety. Crossover was not allowed. Eighty-four patients were randomized to TIL treatment and 84 patients to Ipi. The percentage of patients who had received previous anti-programmed cell death protein 1 therapy was comparable for both study arms (89.3% versus 88.1%). The overall response rate was higher after TIL treatment (48.8% versus 21.4%) and PFS appeared superior as well (HR of progression 0.50, 95% CI 0.35-0.72), but no data were presented to exclude informative censoring imbalance. OS and QoL were presented, although they had not been defined as outcome measures in the NCT02278887 file.^25^ OS data presentation did not encompass information on post-progression treatments per study group and TIL treatment was not shown to deliver a significant OS benefit compared to Ipi. Overall health-related QoL assessed by means of the EORTC Quality of Life Questionnaire Core 15 Palliative Care (QLQ-C15-PAL) was claimed to be superior in the TIL treatment group.^26^ The reported statistically significant difference of 7.7 points at 6 months can however not be regarded as clinically important based on previous validation research.^27^^,^^28^ The percentage of patients with serious TRAEs was higher in the TIL study arm (100% versus 57.3%). It was not reported whether there had been toxic deaths in either study arm. Based on the data presented (HR of progression 0.50, median PFS in the comparator group <6 months, long-term plateau in the PFS curve, >10% improvement in PFS at 1 year), the MCBS grade achieved with TILs is 4. Regarding the applicability of this intervention in daily practice, there are several issues. Firstly, the trial was carried out in two highly specialized oncology centers: the introduction of TIL treatment in other centers may require an intensive learning curve to achieve a comparable safety/risk profile. Secondly, the first-line therapy was not specified and because of this it is unclear whether the second-line Ipi given in the control arm could be regarded as a standard treatment option, specifically for patients with a BRAF v600 mutation (36 out of 84 patients), for whom no prior targeted therapy was allowed.

## Concluding remarks

Our review of 12 selected ESMO 2022 presentations encompassing 10 phase III trials, 1 randomized phase II trial and 1 phase II trial confirms the high-quality standard of current cancer research and the clinical relevance of the research questions answered (Table 2). The presentations provided a clear informational balance of treatment burden and treatment benefit (Table 3). The proportion of studies reporting global QoL and PROs is steadily increasing. However, in none of the reviewed presentations, PFS data were accompanied by information, which could confirm or exclude imbalance between study groups with regard to informative censoring. Informative censoring in a PFS analysis arises when patients are censored for initiation of an effective anticancer treatment before the protocol-defined progression, and these patients are at a different risk for treatment failure than those who continue therapy. Differences in the percentage of patients censored between treatment arms could lead to aberrant PFS results.^29^ In the majority of reviewed presentations, OS data were not accompanied by information that addressed non-desirable crossover and/or imbalances between study groups with regard to post-progression treatment, whereas these factors could seriously influence OS and should be taken into consideration while interpreting OS results.

**Table 2:** Study settings of the 12 reviewed ESMO 2022 trial presentations

Study name	Tumor type	Treatment setting	Assigned treatment Comparator arm	Assigned treatment Intervention arm	Blinded allocation
TROPICS02	HR-positive HER2-negative breast cancer	Advanced, ≥3rd line	Treatment of physicians’ choice	Sacituzumab govitecan	No
MonarcHER	HR-positive Her2-positive breast cancer	Advanced, ≥3rd line	Trastuzumab + investigators’ choice chemotherapy	Abemaciclib/trastuzumab ± fulvestrant	No
CodeBreak200	k*ras*G12C-mutated NSCLC	Advanced, 2nd line	Docetaxel	Sotorasib	No
IPSOS	NSCLC, no EGFR or ALK sensitizing mutation	Advanced, 1st line, not fit for platinum-doublet therapy	Investigators’ choice chemotherapy	Atezolizumab	No
COSMIC313	Intermediate- or poor-risk clear-cell RCC	Advanced, 1st line	Ipilimumab/nivolumab + placebo	Ipilimumab/nivolumab + cabozantinib	Yes
CheckMate 914	RCC	Adjuvant	Placebo	Ipilimumab/nivolumab	Yes
HR-IRI-APC	Pancreatic cancer	Advanced, 2nd line	5FU/LV	5FU/LV + liposomal irinotecan	No
NICHE2	Mismatch repair-deficient colon cancer	Neoadjuvant	No comparator arm	Ipilimumab/nivolumab	No
309/KEYNOTE-775	Endometrial cancer	Advanced, 2nd line	Treatment of physicians’ choice	Lenvatinib/pembrolizumab	No
SOLO1/GOG-3004	BRCA-mutated ovarian cancer	FIGO stage III/IV, responding to platinum-based therapy	Placebo	Olaparib maintenance	Yes
DeFi	Desmoid tumor	Advanced, ≥2nd line	Placebo	Nirogacestat	Yes
TILs in melanoma	Melanoma	Advanced, 1st or 2nd line	Ipilimumab	TILs	No

FIGO, International Federation of Gynecology and Obstetrics; FU, fluorouracil; HER2, human epidermal growth factor receptor 2; HR, hormone receptor; LV, leucovorin; NSCLC, non-small-cell lung cancer; RCC, renal cell cancer; TILs, tumor-infiltrating lymphocytes.

**Table 3:** Outcome measures reported in the 12 reviewed ESMO 2022 trial presentations

Study name	HR of recurrence (I versus C)	ORR (I versus C)	HR of progression (I versus C)	HR of death (I versus C)	QoL (I versus C)	% Serious TRAE (I versus C)	% TR deaths (I versus C)	ESMO-MCBS grading
TROPICS02	NA	21% versus 14%	0.66 (CI 0.53-0.83)	0.79 (CI 0.65-0.96)	Improved	74% versus 60%	2% versus 0%	2
MonarcHER	NA	NR	AT 0.94 (CI 0.64-1.38) AT + F 0.67 (CI 0.45-1.00)	AT 0.84 (CI 0.57-1.23) AT + F 0.71 (CI 0.48-1.05)	Equal	AT 56% versus AT + F 76% versus 54%	NR	1
CodeBreak200	NA	28.1% versus 13.2%	0.66 (CI 0.51-0.86)	1.01 (CI 0.77-1.33)	Improved	40.4% versus 33.1%	0.3% versus 0.6%	2
IPSOS	NA	16.9% versus 7.9%	0.87 (CI 0.70-1.07)	0.78 (CI 0.63-0.97)	Equal	16.3% versus 33.3%	1% versus 2.7%	2
COSMIC313	NA	40.3% versus 36%	0.73 (CI 0.57-0.94)	NR	NP	73% versus 41%	1% versus 1%	1
CheckMate 914	0.92 (CI 0.71-1.19)	NA	NA	NR	NP	28% versus 2%	NR	1
HR-IRI-APC	NA	13% versus 0.7%	0.36 (CI 0.27-0.48)	0.63 (CI 0.48-0.84)	NP	53.1% versus 43.1%	NR	3
NICHE2	NR	NA	NA	NR	NP	3% versus 0%	NR	NA
309/KEYNOTE-775	NA	33.8% versus 14.7%	0.56 (CI 0.48-0.66)	0.65 (CI 0.55-0.77)	Improved	78.8% versus 60.1%	1.5% versus 2.3%	4
SOLO/GOG-3004	NA	NA	0.33 (CI 0.25-0.43)	0.55 (CI 0.40-0.76)	NP	39.6% versus 20%	NR	4
DeFi	NA	41% versus 8%	0.29 (CI 0.15-0.55)	NP	Improved	57% versus 17%	0% versus 0.8%	5
TILs in melanoma	NA	48.8% versus 21.4%	0.50 (CI 0.35-0.72)	0.89 (CI 0.54-1.27)	Unclear	100% versus 57.3%	NR	4

AT, abemaciclib + trastuzumab; C, comparator arm; CI, 95% confidence interval; DFS, disease-free survival; ESMO-MCBS, European Society for Medical Oncology-Magnitude of Clinical Benefit Scale; F, fulvestrant; HR, hazard ratio; I, intervention arm; NA, not applicable; NP, not performed; NR, not reported; ORR, objective response rate; OS, overall survival; PFS, progression-free survival; QoL, quality of life; serious TRAE, treatment-related adverse event graded 3 or higher; TR, treatment-related.

With regard to the implementation of the investigational later-line interventions reported, a crucial question is whether patients treated in daily practice fit well enough in the reported study frames, which only included patients with an ECOG PS of 0 or 1. The same question arises, when the daily practice patient receives different previous treatment than the patients in the reported study. In such cases, subsequent real-world data analysis may exclude an efficacy–effectiveness gap. We calculated the ESMO-MCBS grade of the new interventions tested in the 11 reviewed randomized trial presentations (Table 3) and grades varied from 1 to 5. Grades 4 and 5 are regarded as substantial clinical benefit. In order to keep cancer care affordable and sustainable, it could become necessary not to consider new treatments with an ESMO-MCBS grade below a certain threshold for reimbursement and subsequent implementation in daily practice. In a recently published Canadian study, the association between oncology drug clinical benefit and the time to public reimbursement was evaluated; an ESMO-MCBS grade of only 1 was no barrier for appraisal and a higher score did not relate to faster approval.^30^
